# Who Is Paying the Extinction Debt? Phylogenetic and Functional Structuring on Greek Islands Is Shaped by Sea‐Level Rise Since the Last Glacial Maximum

**DOI:** 10.1002/ece3.73894

**Published:** 2026-06-29

**Authors:** Nathan M. Michielsen, Cyril Hammoud, Johannes Foufopoulos, Kostas Kougioumoutzis, Amandine Vidal‐Hosteng, E. Emiel van Loon, Johannes De Groeve, Kenneth F. Rijsdijk

**Affiliations:** ^1^ Institute for Biodiversity and Ecosystem Dynamics, University of Amsterdam Amsterdam the Netherlands; ^2^ The Environment Institute, School of Biological Sciences, Adelaide University Adelaide South Australia Australia; ^3^ Center for Macroecology, Evolution, and Climate, Globe Institute, University of Copenhagen Copenhagen Ø Denmark; ^4^ Department of Coastal Systems Royal Netherlands Institute for Sea Research Den Burg the Netherlands; ^5^ School for Environment and Sustainability, University of Michigan Ann Arbor Michigan USA; ^6^ Laboratory of Botany, Division of Plant Biology, Department of Biology University of Patras Patras GR Greece; ^7^ Centre de Recherche sur la Biodiversité et l'Environnement, Université de Toulouse Toulouse France; ^8^ Groningen Institute for Evolutionary Life Sciences, University of Groningen Groningen the Netherlands

**Keywords:** community ecology, island biogeography, landbridge islands, paleogeography, squamates

## Abstract

Since the Last Glacial Maximum (LGM) ~21.5 kya, global sea‐level rise has reshaped coastal areas by contracting subaerial regions, severing mainland connections and driving the progressive fragmentation of pre‐existing islands. These changes led to supersaturation of island communities, triggering community relaxation through local extinctions. However, it remains unclear which ecological processes are responsible for patterns of species extinction. Here, we integrate a paleo‐coastline model with well‐characterized squamate community data from 163 Mediterranean islands to assess how past geographical changes influenced community structure. We evaluate phylogenetic and functional community structure and test links to paleogeographic variables. Our findings suggest phylogenetic overdispersion (co‐occurring species being more distantly related than expected by chance) dominates and is higher on older islands, implying ongoing community relaxation and that extinctions are driven more by interspecific competition than environmental filtering. Island time‐since‐isolation emerges as the strongest predictor of phylogenetic structuring, indicating that longer isolation drives phylogenetic overdispersion via selective extinction of close relatives. These results underline the significance of relaxation dynamics in shaping insular communities.

## Graphical abstract

Since the Last Glacial Maximum, sea‐level rise has isolated, fragmented, and shrunk islands, supersaturating squamate communities and triggering ongoing community relaxation. Integrating a paleo‐coastline model with community data from 163 islands, we show that phylogenetic overdispersion dominates insular squamate communities and increases with island age, consistent with interspecific competition, rather than environmental filtering, as the primary driver of extinction. Island time‐since‐isolation emerges as the strongest predictor of community structure, highlighting the lasting imprint of post‐glacial geographic change on present‐day biodiversity.

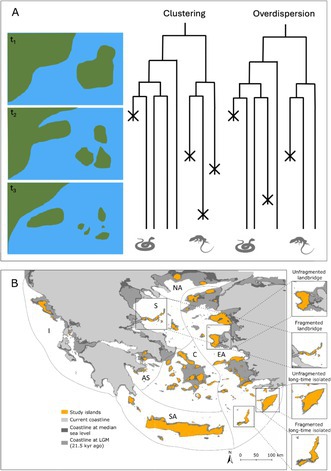

## 1 Introduction

Islands are ideal model systems to study the biogeographic processes that shape the emergence, distribution, and diversity of species due to their discrete nature, variable geographic properties, and highly diverse biota (Darwin 1859; Whittaker et al. 2017). The field of island biogeography increasingly recognizes islands' idiosyncratic and dynamic physical, environmental, and ecological characteristics through time as factors that shape local species communities (Fernández‐Palacios et al. 2016; Whittaker et al. 2008). Since the Last Glacial Maximum (LGM, ~21.5 kyr ago), global eustatic sea‐levels have risen ~130 m (Foufopoulos and Ives 1999; Lambeck et al. 2014; Simaiakis et al. 2017), causing coastlines to recede, insular areas to shrink, islands to fragment, and landbridges to drown (Ali and Aitchison 2014; Fernández‐Palacios et al. 2016; Hammoud et al. 2021; Rijsdijk et al. 2014; Simaiakis et al. 2017). How insular communities responded to sea‐level rise depended on island geography since the magnitude, rate, and chronology of geographical changes varied widely (Fernández‐Palacios et al. 2016; Simaiakis et al. 2017).

For organisms with low dispersal capability, equilibrium theory predicts that landbridge islands, that is, islands that were formerly connected to the mainland during periods of lower sea level and became isolated as sea levels subsequently rose, contain higher biodiversity than expected by their area alone, a hypothesis corroborated by empirical evidence (Diamond 1972; Hammoud et al. 2021; Wilcox 1978). Supersaturation, the condition in which a community's species richness exceeds what would be expected based on island area alone, may also occur on non‐landbridge islands that have rapidly decreased in area, whether through progressive shrinking or, particularly, through fragmentation (Fernández‐Palacios et al. 2016; Hammoud et al. 2021; MacArthur and Wilson 1967; Simaiakis et al. 2017). This excess diversity is typically rectified through community relaxation: the process that causes the gradual reduction of species richness by (local) extinction (Figueiredo et al. 2019). Especially for poor‐dispersing species, the resulting relaxation faunas (supersaturated communities undergoing a delayed decline in species richness) are primarily shaped by local extinctions occurring over long periods of time (Cardillo et al. 2008; Diamond 1972; Wilcox 1978). Despite growing interest in the future loss of biodiversity due to time‐lagged responses to environmental change or disturbance, a phenomenon known as extinction debt (Figueiredo et al. 2019; Halley et al. 2016; Kuussaari et al. 2009; Ramírez et al. 2023), relaxation dynamics over millennial timescales remain poorly understood, in particular whether these relaxation extinctions are random or deterministic with regard to species' phylogeny and functional ecology. This information can help us to understand the ecological processes that play a role in “collecting the extinction debt” (Kraft et al. 2007; Webb et al. 2002).

**FIGURE 1:**
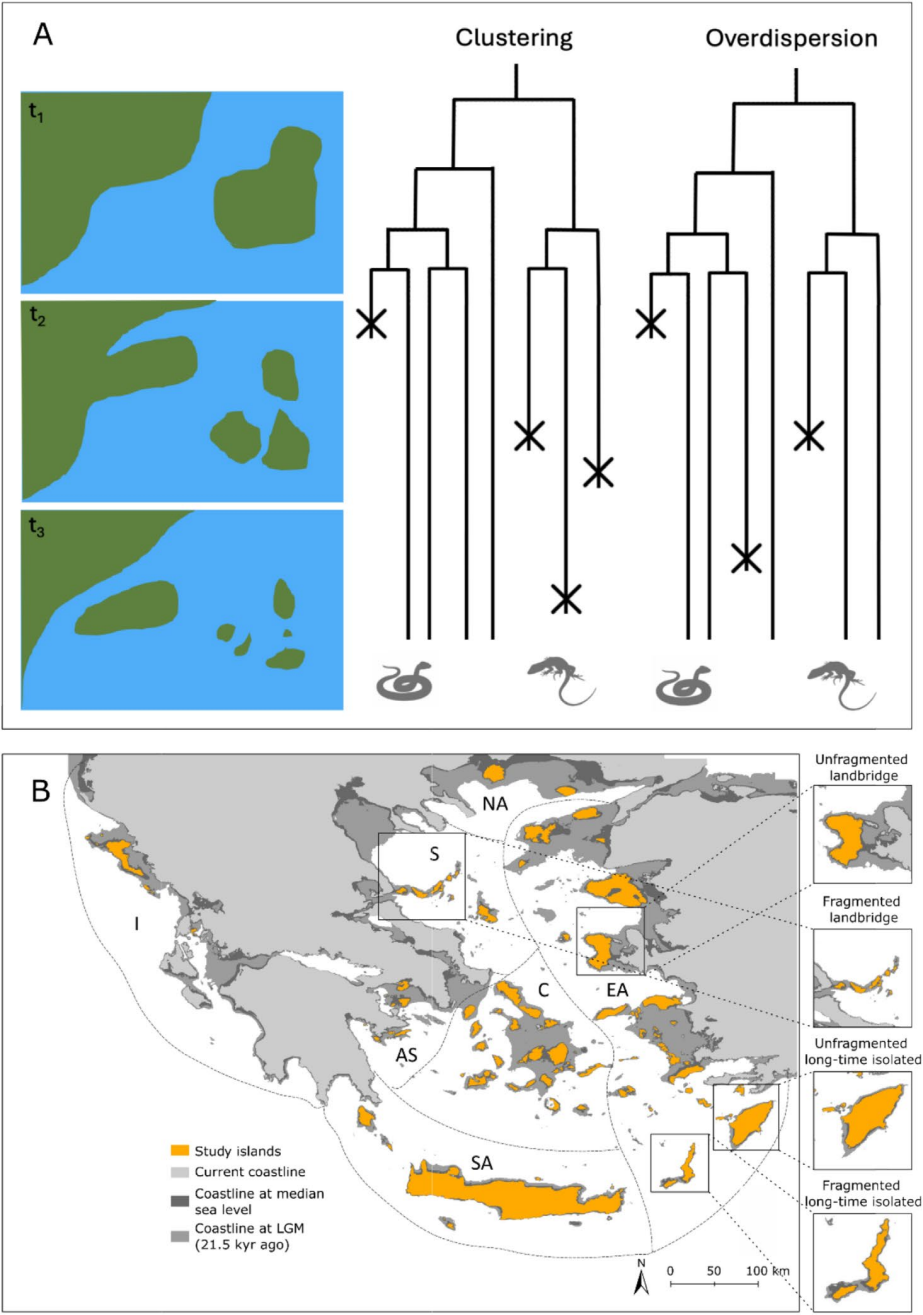
Schematic representation of hypotheses and geographic extent of study. (A) Schematic of potential community structuring following landbridge formation and island fragmentation due to sea‐level rise. Phylogenies depict community composition over time. Initially (t_1_), mainland and island communities are at equilibrium. After isolation (t_2_), communities become supersaturated. Over time (t_3_), selective extinction leads to functional and phylogenetic overdispersion (right) or clustering (left). Extinctions are shown by crossed‐out and truncated branches; surviving species reach the bottom of each phylogeny. (B) Map of the Aegean and Ionian archipelagos showing current geography and paleo‐coastlines (De Groeve et al. 2025) at the Last Glacial Maximum (21.5 kya) and median sea level over the last 9 glacial cycles (65 m below current sea‐level). Study islands are shaded in orange; biogeographic subregions are outlined with dotted lines: AS = Argo‐Saronic (*n* = 20), C = Cyclades (*n* = 65), EA = East‐Aegean (*n* = 39), I = Ionian (*n* = 13), NA = North‐Aegean (*n* = 2), SA = South‐Aegean (*n* = 6), S = Sporades (*n* = 18). Inset maps show examples of island types: Unfragmented landbridge (Chios), Fragmented landbridge (Sporades archipelago: Skiathos, Skopelos, Alonnisos, Peristera, Kira Panagia, Gioura, Psathoura), Unfragmented long‐time isolated (Rhodes), Fragmented long‐time isolated (Karpathos, Kasos).

The contemporary structure of ecological communities of species with poor colonizing ability is directly influenced by past extinctions (Brown 1971; Foufopoulos et al. 2011). Therefore, patterns of taxonomic, phylogenetic and functional community structure have been used to explore the contribution of different community assembly processes, such as environmental filtering (Graham et al. 2009; Lebrija‐Trejos et al. 2010; Sutton et al. 2021) or competitive exclusion (Cardillo 2011; Case 1983; Graham et al. 2009), in shaping ecological communities. A central assumption underlying such inferences is that extinction is non‐random with respect to species traits, and that the functional traits considered are phylogenetically conserved. Under environmental filtering, extinctions tend to remove species with traits poorly suited to the environment, resulting in communities of closely related species with similar successful traits—leaving a signature of phylogenetic and functional clustering (Figure 1A). Alternatively, closely related and ecologically similar species may compete more strongly than distantly related and functionally dissimilar species. Thus, under competitive exclusion, extinctions would preferentially target species with overlapping traits, producing phylogenetically and functionally overdispersed communities (Figure 1A) (Haydon et al. 1993; Webb 2000; Webb et al. 2002). Although phenotypic convergence among distantly related species has the potential to decouple functional and phylogenetic patterns, in our study system key traits appear largely conserved, limiting this effect.

Here, we investigate for the first time whether the community structure of insular relaxation faunas is linked to paleogeographic changes, by exploring how community structure is shaped by land contraction, island fragmentation and isolation from the mainland by rising sea‐levels since the LGM. We begin by implementing a new, high‐resolution coastline model to characterize island fragmentation and infer timings of isolation of 163 Greek islands, which responded heterogeneously to post‐LGM sea‐level rise (Hammoud et al. 2021; Simaiakis et al. 2017). We then use a detailed and resolved squamate (Order: Squamata) community dataset (Present Day and Paleo Ecological Distributions of Reptiles in the Aegean and Ionian Seas 2023) to characterize taxonomic diversity and phylogenetic and functional community structure. Finally, we test whether the phylogenetic and functional structure of these communities is random or deterministic using the Net Relatedness Index (NRI), and examine its relationship to paleogeographic variables using multivariate linear models. We conclude by discussing the implications of the patterns of community structuring on relaxation dynamics and the conservation of island biota.

## 2 Methods

### 2.1 Study System

The Greek islands form a vast archipelago, comprising over 3500 islands across seven biogeographic subregions with a shared fragmentation history (Blondel et al. 2010; Foufopoulos and Ives 1999; Poulakakis et al. 2015) (Figure 1B). This is an ideal system to investigate post‐LGM faunal relaxation, having been extensively studied by ecologists and biogeographers (Sfenthourakis and Triantis 2017; Wettstein 1957), and exhibiting diverse geographic responses to sea‐level rise (Simaiakis et al. 2017). The archipelago includes long‐time isolated continental islands (sensu Ali 2017, e.g., Crete), as well as recently formed (mostly < 15 ky ago) landbridge islands that were formerly connected to the European (e.g., the Argo‐Saronic Islands) or Asian mainland (e.g., parts of the Dodecanese). Some islands fragmented into multiple landmasses in response to sea‐level rise, while others remained singular but shrank (Hammoud et al. 2021; Simaiakis et al. 2017). Our study focuses on islands and paleo‐islands spanning this paleogeographic diversity (Figure 1B, described in detail in Section 2.2), and for which complete squamate community inventories are available (see Section 2.3).

Squamates are well‐suited to study extinction dynamics in this region due to their high species richness of 51 species across all islands (Valakos et al. 2008; Present Day and Paleo Ecological Distributions of Reptiles in the Aegean and Ionian Seas 2023), and well‐samples diversity, with detailed inventories for many islands (Foufopoulos et al. 2011; Lymberakis et al. 2018; Valakos et al. 2008), and readily available phylogenetic (Tonini et al. 2016) and functional trait data (Feldman et al. 2016; Valakos et al. 2008). Given the very limited over‐water dispersal abilities of Greek insular squamates (Foufopoulos et al. 2011; Hammoud et al. 2021), due to the region's comparatively cold sea temperatures and lack of floating vegetation (Hurston et al. 2009), successful natural dispersal events are highly improbable, even across the region's sometimes narrow stretches of shallow sea. Consequently, inter‐island dispersal is unlikely to influence present‐day community assembly. The squamate communities span lizards (genera *Podarcis*, *Lacerta*, *Mediodactylus*) and snakes (genera *Dolichophis*, *Elaphe*, *Natrix*, *Vipera*), with composition varying across island clusters according to the nearest mainland source area. The region also harbors a pronounced endemic element (e.g., the lacertid lizards *Podarcis milensis*, *P. cretensis* , *P. gaigeae* , and the geckonid *Mediodactylus oertzeni*; as well as the snakes *Macrovipera schweizeri* and *Dolichophis jugularis zinneri*) however, as perhaps expected, these taxa occur on pre‐Pleistocene, deep‐water islands (see Supporting Information for a more detailed description of the natural history of Aegean squamate communities).

### 2.2 Study Islands and Paleogeographic Categorization

The study's geographic extent covers 163 islands and islets in the Aegean and Ionian Seas (Figure 1B and Table S1), including 116 extant and 47 paleo‐islands, hereafter referred to as “study islands.” Paleo‐islands were pre‐fragmented combinations of currently extant islands that existed at any point since the LGM. Their inclusion provides insight into communities' past biodiversity changes. Paleocommunity composition is inferred under the assumption that differences between island fragment communities arise solely through local extinction, with each fragment initially inheriting the full assemblage present on the parent landmass (Foufopoulos et al. 2011). Study islands are grouped into seven biogeographic subregions (Figure 1B and Data S1, Blondel et al. 2010; Foufopoulos and Ives 1999; Poulakakis et al. 2015). We selected study islands based on high‐quality reptile inventories and a minimum of two squamate species per island, the minimal number required to calculate phylogenetic and functional community structure.

We used a paleogeographic coastline reconstruction (see https://doi.org/10.21942/uva.c.6678104.v1), generated with a development version of the *tabs* R package, following the workflow proposed by De Groeve et al. (2025). Specifically, the coastline was reconstructed by classifying grid cells from a bathymetric–topographic model (115 × 115 m) according to their elevation relative to past sea level. Sea level estimates are locally adjusted for uplift and subsidence. Cells above this corrected sea level are classified as terrestrial and those below as marine. The resulting land–sea boundary defines a temporally consistent coastline that accounts for tectonic processes in the Aegean region. The high‐resolution bathymetric–topographic model consists of 44 coastline rasters spanning from the LGM (21.5 kya) to the present, at 500‐year intervals. From these, we extracted fragmentation timing and eight (paleo)geographic variables (Table S1, Data S1). We categorized the 163 study islands into four types based on pre‐LGM mainland connection (paleopeninsularity) and post‐LGM fragmentation (Table S2): fragmented and unfragmented landbridge islands and fragmented and unfragmented long‐time isolated islands. We classified continental‐shelf islands (sensu Ali 2017) that were connected to the European or Asian mainland during the LGM as landbridge islands. If landbridge islands fragmented after isolation, they were classified as fragmented landbridge islands; if not, they were classified as unfragmented landbridge islands. We applied the same fragmentation‐based classification to continental‐shelf islands that were isolated throughout glacial cycles, labeling them as fragmented or unfragmented long‐time isolated islands. We also grouped islands into seven biogeographic subregions, defined here as groups of islands that form major archipelagos and share a common fragmentation history (Figure 1B, Table S1). For each island, we estimated current and LGM paleo‐area, percentage of area lost, and time‐since‐isolation (Table 1). Unfragmented long‐time isolated islands have been isolated far longer than other island types. To account for this, we assigned them a counterfactual isolation time of 21.5 ky (the maximum in the study's timeframe) and a binary pre‐LGM isolation indicator. We tested whether pre‐LGM isolation altered temporal isolation effects using an interaction term in our OLS model. We did not include distance to nearest landmass, as previous studies found no effect of spatial isolation on Greek island herpetofauna, likely due to their poor overwater dispersal ability (Foufopoulos et al. 2011; Hammoud et al. 2021; Hurston et al. 2009).

**TABLE 1:** Summary of all predictor variables.

Predictor variable	Abbreviation	Description	Unit
Island type	IT	Classification of study islands based on the combination of paleopeninsularity defined as connection to the mainland during the LGM and fragmentation as a result of post‐LGM sea‐level rise	Categorical
Biogeographic subregion	BR	Classification of study islands into one of seven distinct biogeographic subregions of Greek islands	Categorical
Area	A	Current area of study islands	m^2^
Paleo‐area	PA	Area of an island at its earliest reconstructed timestep, representing either the area of a currently existing island at its emergence, or the combined area of a group of present‐day islands before they became fragmented	m^2^
Area change	ΔA	Absolute change in island area from the first timestep until the present (extant islands) or until fragmentation (paleo‐islands)	m^2^
Percentage of area change	ΔA%	Percentage of area that was lost with fragmentation or since its emergence until the present	% of paleo‐area
Rate of area change	ΔAR	The rate of area decrease	m^2^/yr
Time‐since‐isolation	TSI	The time between the first timestep of an island's existence until now or until the last timestep before further fragmentation	yr

*Note:* Table includes all extracted (paleo)geographic variables for all study islands from paleo‐coastline polygons reconstructed using the TABS model from De Groeve et al. (2025). Table includes descriptions, abbreviations, and units per predictor variable.

### 2.3 Biodiversity Data

We compiled species inventories for all study islands from the herpetofaunal literature (primarily; Valakos et al. 2008) and data from 30 years of field work (Foufopoulos 2023, data available at: https://doi.org/10.21942/uva.23284868.v2). There is evidence of potential seafaring between Greek islands up to as early as 30,000 BC (Carter et al. 2019), giving opportunities for naturally poor‐dispersing squamate species to anthropogenically spread across islands, which would affect phylogenetic and functional community structure patterns (Cadotte et al. 2019). Around 20% of Greek insular squamate species (10 out of 52) show evidence of anthropogenic introduction and establishment (*Ablepharus kitaibelii*, *Anatololacerta* sp. complex, *Chalcides ocellatus*, *Chamaeleo chamaeleon* , *Eryx jaculus* , *Hemidactylus turcicus* , *Laudakia stellio*, *Tarentola mauretanica*, *Telescopus fallax* , and *Zamenis situla* ). Evidently anthropogenically dispersed species were removed from species lists (Data S1).

Six out of 51 species in the community matrix represent species complexes which likely contain multiple cryptic species under the same taxon name. These instances including references are listed in Data S2. Future genetic analyses may revise taxonomy, but we follow the taxonomy used in Tonini et al. (2016) and Feldman et al. (2016), which do not recognize these complexes. We deem it justified to incorporate these taxa, since closely related species are unlikely to be significantly phylogenetically or functionally different.

### 2.4 Phylogenetic and Functional Structure

We extracted the assemblage‐level phylogenies of all insular squamate communities to calculate phylogenetic diversity (PD) metrics using the *ape* (Paradis and Schliep 2019) and *phytools* R packages (Revell 2012). We pruned the maximum clade credibility (MCC) consensus tree from Tonini et al. (2016), which summarizes a posterior distribution of 10,000 trees with branch lengths reflecting median divergence times, to include only species present on each island. Of the 43 species included in phylogenetic analyses, 41 (95.3%) have molecular data supporting their placement in the phylogeny, with only *Anatololacerta pelasgiana* and *Malpolon insignitus* placed on the basis of taxonomic information alone. We pruned the high‐quality squamate phylogeny (Tonini et al. 2016) to include only species present on each island. We then calculated the phylogenetic Mean Pairwise Distance (MPD) for each assemblage using the *picante* package (Kembel et al. 2010). MPD measures the average evolutionary or functional distance between species, with high MPD indicating more unrelated or dissimilar species. We used MPD over other pairwise metrics (e.g., MNTD) for easier interpretation, especially for FD (Weiher and Keddy 1995). MPD is also less sensitive to uneven species distribution across clades, which is important for detecting clustering, and better captures environmental filtering via its derived Net Relatedness Index (NRI) (Kraft et al. 2007; Vellend et al. 2011). We used these phylogenetic distance matrices to calculate the MPD for all communities.

As in Ramm et al. (2018), we use body size and microhabitat preferences (primary zone of activity), as functional traits (Data S2). These traits capture functional diversity (FD), as species with similar body sizes and microhabitat preferences are more likely to be functionally similar and compete for resources (Ramm et al. 2018). For example: although *Ablepharus kitaibelii* (snake‐eyed skink) and *Mediodactylus kotschyi* (Kotschy's gecko) have similar body sizes (3.7 and 3.8 g, respectively, Data S2), they differ in microhabitat usage with *A. kitaibelii* being a ground‐dweller among leaf litter and low vegetation and *M. kotschyi* preferring a rocky substrate (terrestrial and saxicolous, respectively, Data S2), meaning it is unlikely they compete for the same resources. On the other hand, *M. kotschyi* and *Heremites auratus* (levant skink) occupy the same microhabitat (both saxicolous, Data S2), but differ greatly in body size (body size = 3.8 and 33.8 g, respectively, Data S2), reducing competition. However, *Podarcis tauricus* and *P. erhardii*, which are similarly sized (13.7 and 17.3 g) and occupy the same habitat (terrestrial and terrestrial/saxicolous, respectively, Data S2), are more likely to compete.

We gathered species' body sizes from Feldman et al. (2016) and grouped their habitat preferences into 10 categories based on their primary zone of activity (Ramm et al. 2018): terrestrial, fossorial, generalist, arboreal, saxicolous, aquatic and combinations of these, which we treated as distinct categories. The herpetological literature (primarily Valakos et al. 2008, alternative sources listed in Data S2) sourced species' microhabitat preferences, which were further validated by extensive field observations by JF. The spatial distribution of both traits across contemporary study islands is illustrated in Figure S2. Following standard convention in the field, we log‐transform all species body size data. We did not find body size and microhabitat preference to covary. We tested for phylogenetic signal of body size using the *phylosignal* package in R (Keck et al. 2016) (Table S5) to check whether functional traits are phylogenetically conserved, a prerequisite for interpretation of phylogenetic and functional community structuring (Kamilar et al. 2015; Webb 2000). Since no similar test exists for categorical traits, we did not test for a phylogenetic signal of microhabitat preference. Since a change in microhabitat preference likely requires significant evolutionary investment, we consider it phylogenetically conserved within our species pool. We calculated functional dissimilarity using Gower's distance (Gower 1966), suitable for mixed continuous and categorical traits (Barton and Barton 2015), and derived functional distance matrices per island to calculate functional MPDs.

In summary, both phylogenetic and functional diversity in this study are quantified through the Mean Pairwise Distance (MPD) framework, applied separately to phylogenetic and functional distance matrices. Phylogenetic MPD captures the average evolutionary relatedness among species in a community, while functional MPD captures the average dissimilarity in body size and microhabitat preference. To contextualize these raw diversity values relative to a null expectation, we derive the Net Relatedness Index (NRI) from each MPD, which standardizes observed MPD against a null distribution of randomly assembled communities of the same richness. Together, MPD describes the magnitude of phylogenetic or functional diversity within a community, while NRI describes its structure relative to the regional species pool, allowing us to distinguish not just how diverse communities are, but whether their composition departs from random expectation in a biologically meaningful direction.

### 2.5 NRI and Community Structure

We calculated the phylogenetic and functional Net Relatedness Index (NRI; Webb et al. 2002) for all island communities. NRIs indicate assemblage structure (or lack thereof) and show if observed structuring is significantly non‐random by comparing observed MPD values to an ecologically relevant null‐distribution of subsamples. We assembled null communities by randomly shuffling taxon labels of squamate species across islands for 999 iterations. We thus assume the complete collection of Aegean and Ionian insular squamates as the source pool of species that can establish on islands.

Negative NRIs and low quantiles (*p* < 0.025) indicate significant phylogenetic or functional overdispersion, while positive NRIs and high quantiles (*p* > 0.975) indicate significant phylogenetic or functional clustering (Kembel et al. 2010; Webb et al. 2002). Communities within the null distribution are not significantly structured, but NRI values still provide comparative insight (e.g., an NRI of 1 is more clustered than −1, even if neither is significant) (Kamilar et al. 2015). Therefore, we used phylogenetic and functional NRI as a continuous predictor across islands, regardless of significance. We then determined the proportions of different types of structuring per diversity metric (PD and FD) (i) across island types, (ii) between all landbridge and long‐time isolated islands, and finally (iii) between all fragmented and unfragmented islands.

### 2.6 Statistical Analyses

As preliminary exploration, we performed univariate Ordinary Least Squares (OLS) regression between diversity metrics and predictor variables. For area and paleo‐area, we first fitted Island Species Area Relationships (ISARs) using three models: linear, Arrhenius power, and exponential functions. We chose these models since they most often provide an adequate fit, with optimal explanatory power for ISARs (Triantis et al. 2012). We compared ISARs using AIC and adjusted *R* ^2^ values. Arrhenius power functions best described species richness, while exponential functions fit phylogenetic and functional diversity area relationships (Table S2). Thus, we used Arrhenius power functions for consequent multivariate species richness models and we log‐transformed area and paleo‐area in phylogenetic and functional diversity models. We assessed predictor covariation using the R package “corrplot” (Wei et al. 2017) before inferential multivariate analyses (Figure S3). We excluded collinear variables (rho < 0.7) and variables that caused model instability. Specifically, we removed area change and rate of area change, due to covariation with paleo‐area (Figure S3). We retained paleo‐area in global models, as it likely influences the difference in area and the rate of area change, rather than the reverse.

After these preliminary steps, we assessed whether (paleo)geography is linked to the taxonomic diversity and phylogenetic and functional structure of squamate communities. We fitted three global multivariate models by regressing insular species richness, PD NRI and FD NRI, on all remaining variables that showed a significant univariate relationship with the respective diversity metric (island type; biogeographic subregion; log(area); log(paleo‐area); percentage of area change; time‐since‐isolation). Using the “dredge” function (MuMIn package, R, Barton and Barton 2015), we selected models with the lowest AIC (ΔAIC < 2) and highest adjusted *R* ^2^.

We tested for interactive effects between area and time‐since‐isolation, hypothesizing that area effects differ between islands that have been isolated the longest (i.e., long‐time isolated and unfragmented islands) and islands that more recently became isolated (i.e., landbridge and fragmented islands). No significant interaction was found, and no other interactions were evaluated. We used a binary indicator for pre‐LGM isolation (TRUE for unfragmented long‐time isolated islands) to assess if those islands are influenced by time‐since‐isolation differently, but since no significantly different relationship was found, time‐since‐isolation was kept at 21.5 kyr for these islands (see Section 2.2). We conducted *t*‐tests comparing PD and FD NRI between landbridge versus long‐time isolated islands and fragmented versus unfragmented islands. Significant categorical predictors in the final models were further analysed using ANOVA with Tukey HSD post hoc tests.

## 3 Results

### 3.1 Paleogeography and Community Structuring

Island types, defined by the combination of paleopeninsularity and fragmentation history, are unevenly distributed across the archipelago. Fragmented long‐time isolated islands (*n* = 69) and fragmented landbridge islands (*n* = 63) are much more common than their unfragmented counterparts (*n* = 22 and *n* = 9, respectively; Figure S1, Table S1). Considering the two factors individually, fragmented islands far outnumber unfragmented ones (132 vs. 31; Figure S1, Table S1), while landbridge and long‐time isolated islands are more evenly represented (72 vs. 91; Figure S1, Table S1).

**FIGURE 2:**
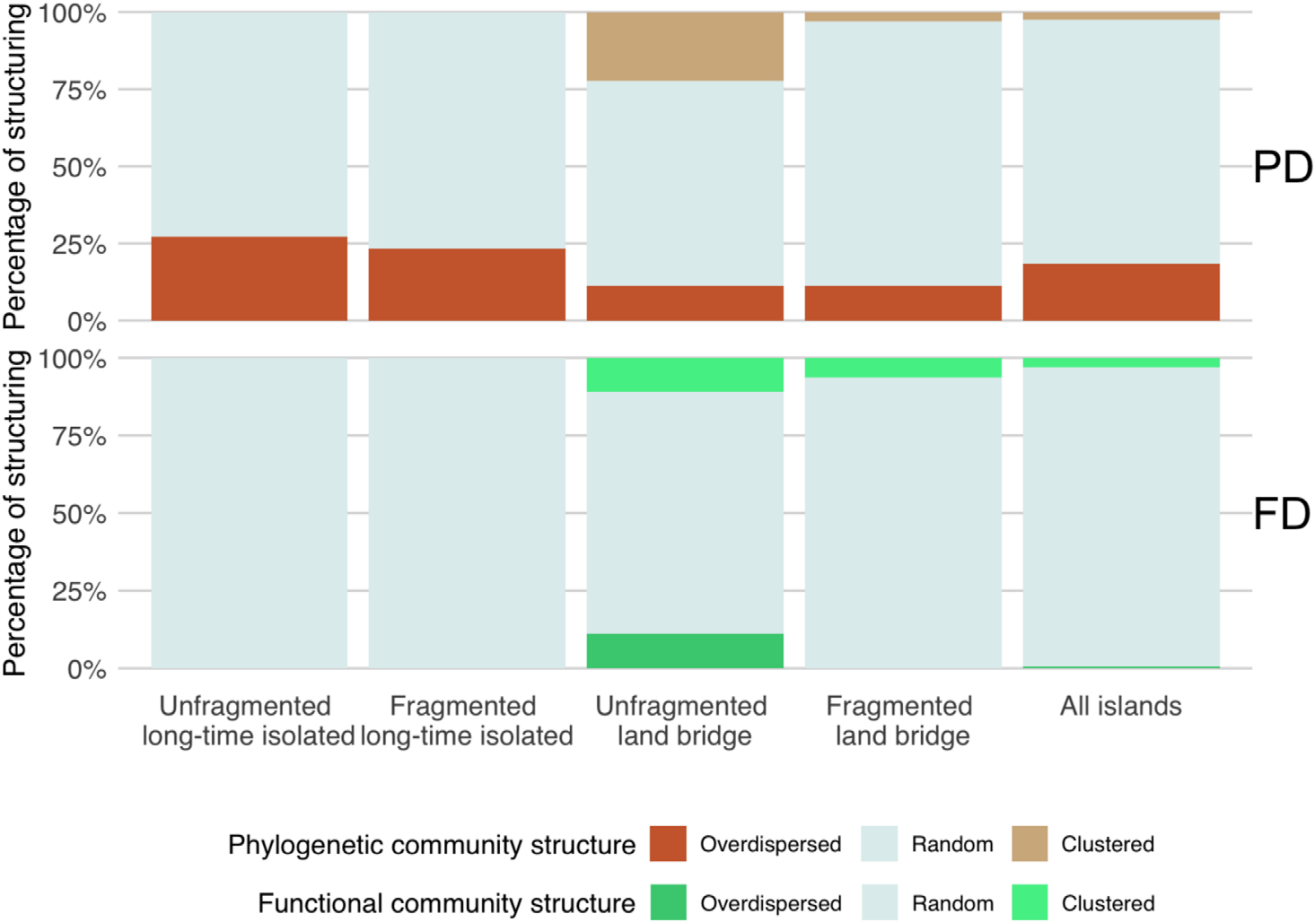
Phylogenetic and functional community structuring per island type. Percentages of islands with significantly overdispersed, clustered, or randomly structured communities with respect to phylogenetic (PD) and functional diversity (FD). Significance of community structure tested using ecological null models comparing PD and FD of extant communities with 999 null communities of the same size, with randomly shuffled taxa labels. Bars represent island types: Unfragmented long‐time isolated (*n* = 22), fragmented long‐time isolated (*n* = 69), unfragmented landbridge (*n* = 9), fragmented landbridge (*n* = 63), and all islands (*n* = 163).

**FIGURE 3:**
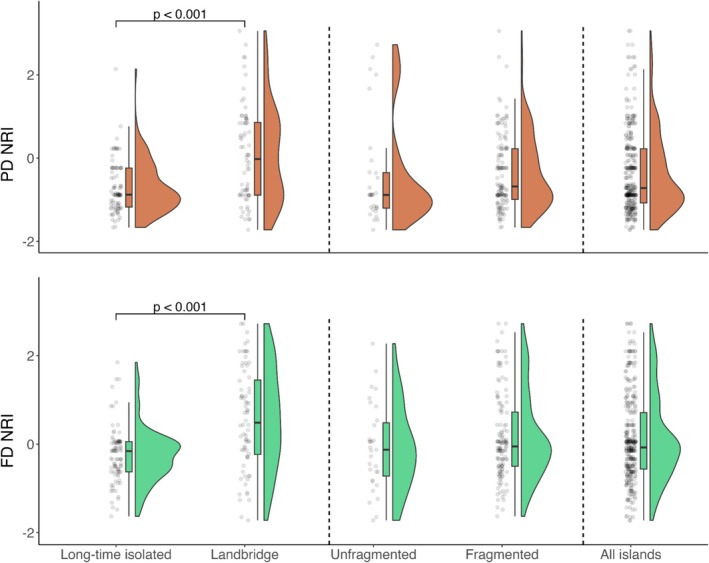
Phylogenetic and functional NRI per category of paleopeninsularity and fragmentation history. Distributions of PD (orange) and FD NRI (green) per category of paleopeninsularity: Long‐time isolated (*n* = 91) and landbridge (*n* = 72), and per category of fragmentation: Unfragmented (*n* = 132) and fragmented (*n* = 31). Categories between vertical dotted lines contain data of all islands. *p*‐values denote significant differences.

Squamate diversity across the Greek islands considered here varies widely in species richness (2–21 species), phylogenetic diversity (PD NRI: −1.7 to 3.1), and functional diversity (FD NRI: −1.7 to 2.7) (Figure S1, Table S1). Most communities (70.6%) tend toward phylogenetic overdispersion (negative PD NRI; Figures 2 and 3), with 18.4% significantly overdispersed and 2.5% significantly clustered. However, the majority show no significant structure in PD (79.1%) or FD (96.3%) (Figure 2). For FD, significant clustering (3.1%) is more common than overdispersion (0.6%).

The proportion of significantly structured communities varies by island type and the type of structuring considered (Figure 2). The highest proportions of phylogenetically overdispersed communities occur on fragmented long‐time‐isolated islands (23.2%) and unfragmented long‐time‐isolated islands (27.3%) (Figure 2). In contrast, fragmented and unfragmented landbridge islands exhibit significantly lower proportions of phylogenetic overdispersion (both 11.1%) (Figure 2, Table S1). Phylogenetic clustering is absent on long‐time‐isolated islands but observed on unfragmented landbridge islands (22.2%, inflated by a small sample size, *n* = 9, Table S1) and, to a lesser extent, fragmented landbridge islands (3.2%) (Figure 2). For functional community structure, no significant structuring is detected on long‐time‐isolated islands, while landbridge islands show some structuring. Unfragmented landbridge islands exhibit a higher degree of functional clustering (11.1%) than fragmented landbridge islands (6.3%) (Figure 2).

### 3.2 OLS Models

**FIGURE 4:**
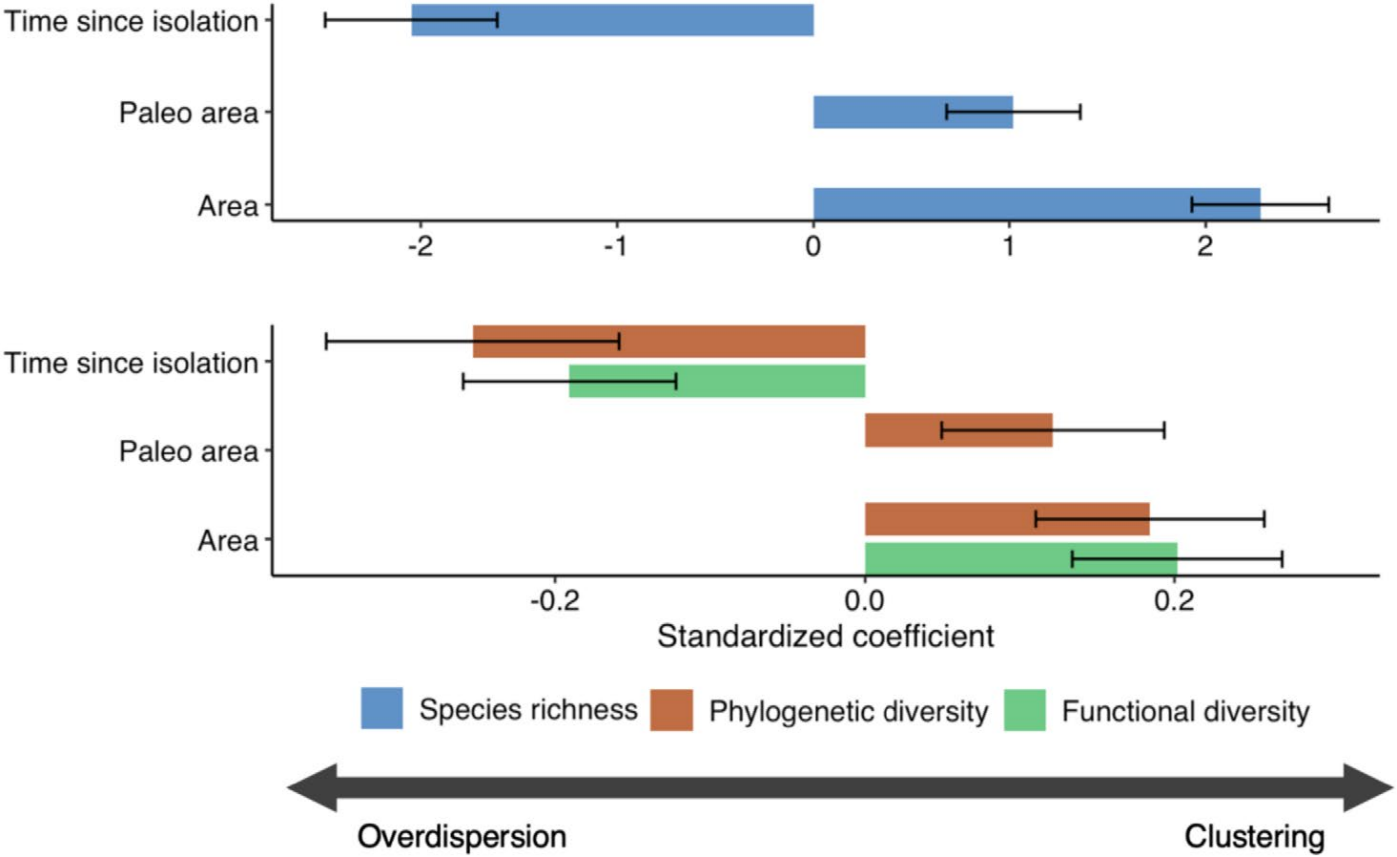
Coefficients of selected models. Horizontal colored bars and whiskers indicate the direction and scaled effect size (± standard error) of significant continuous predictors from the preferred OLS models (Table 2) for each diversity metric. Arrows indicate increasing phylogenetic and functional overdispersion or clustering. Coefficients associated with the categorical “island type” variable are reported in Figure S5.

OLS model selection reveals that PD NRI and FD NRI are associated with several of the islands' (paleo)geographic characteristics, including time‐since‐isolation (Figure 4 and Table 1). The preferred PD NRI model includes island type, biogeographic subregion, area, paleo‐area, and time‐since‐isolation (Figure 4 and Table 1). Time‐since isolation is the only variable that has a negative effect on PD NRI. Together, these variables explain 45% of the variance (Table 1). The preferred FD NRI model includes island type and time‐since‐isolation, which collectively explain 32% of the variance (Table 1). The insular species richness (SR) model, similar to the PD NRI model, includes island type, biogeographic subregion, area, paleo‐area, and time‐since‐isolation, with time‐since‐isolation again having a negative effect. Collectively, these variables explain 58% of the total variance.

The models reveal a pattern of increasing phylogenetic clustering (increasing PD NRI) and SR with increasing area and paleo‐area (Figure 4 and Table 2). However, as time‐since‐isolation of fragmented and landbridge islands increases, phylogenetic and functional overdispersion increases (PD and FD NRI decrease) and species richness declines (Figure 4 and Table 2). Squamate communities on landbridge islands also exhibit significantly higher NRI values than those on islands that have remained isolated since before the LGM, suggesting lower levels of overdispersion on landbridge islands (Figure 3 and Tables S1, S3, and S4). Fragmented islands similarly have higher PD and FD NRI values than unfragmented islands, although this difference is not statistically significant (Figure 3 and Tables S1 and S3).

**TABLE 2:** Top performing models' structure, coefficients, and performance.

Model	IT	BR	A	PA	ΔA%	TSI	df	ΔAIC	Adj. R^2^
PD1*	**+IT**	**+BR**	**+0.18 log(A)**	**+0.12 log(PA)**		**−0.25 TSI**	**14**	**0**	**0.45**
PD2	+IT	+BR	+0.24 log(A)			−0.24 TSI	13	0.70	0.40
FD1*		**+BR**	**+0.20 log(A)**			−**0.19 TSI**	**10**	**0**	**0.32**
**SR1**	**+IT**	**+BR**	**+2.28A**	**+1.02 PA**		**−2.04 TSI**	**14**	**0**	**0.58**
SR2		+BR	+2.53 A	+0.88 PA		−1.53 TSI	11	1.32	0.57
SR3*	+IT		+2.53 A	+0.98 PA		−1.89 TSI	8	1.87	0.59
SR4	+IT	+BR	+2.53 A	+0.80 PA	+0.32 ΔA%	−2.07 TSI	15	1.88	0.58

*Note:* Summary of model structures, estimated scaled coefficients, df, ΔAIC and adjusted *R* ^2^ of top performing OLS models. Empty cells indicate variables were not included in the respective models. Continuous variables include estimated scaled coefficients. The table presents all models with ΔAIC < 2. These models are selected from all possible permutations using the variables included in the full models. Selected models are in bold. Selected models indicated by asterisks. IT = Island type, BR = biogeographic subregion, A = area, PA = paleo‐area, ΔA% = percentage of area change, TSI = time‐since‐isolation (Table 1). Description of predictor variables can be found in the methods section. All variables included in final models are statistically significant (*p* < 0.05).

## 4 Discussion

Squamate communities on Greek islands show patterns of significant community structuring linked to geographic changes from sea‐level rise since the last glacial period. Here, we hypothesize that processes like landbridge submersion, island fragmentation, and area reduction affect community composition and structure. Hence, we set out to explore the legacies that selective relaxation, demonstrated in previous studies (Foufopoulos et al. 2011), have left in the contemporary phylogenetic and functional structure of reptiles on 163 islands by producing models that describe how community structure relates to response to paleogeographic change caused by post‐LGM sea‐level rise.

Our data show that squamate communities on islands in the Aegean and Ionian Seas exhibit predominantly phylogenetic overdispersion rather than clustering. In other words, species in island communities are generally less evolutionarily related than would be expected by chance. By contrast, patterns of functional structure are weaker and less consistent across islands. These emerging patterns are closely linked to the region's dynamic geographical changes caused by sea‐level rise since the end of the last glacial period. Landbridge islands contain relaxation faunas that are less phylogenetically overdispersed than older islands that have been isolated throughout glacial–interglacial cycles. Fragmented islands show a similar, though non‐significant, pattern. Furthermore, the time an island has been isolated is the most important predictor of phylogenetic diversity, positively associated with phylogenetic overdispersion and with an effect exceeding the usual suspect of island biogeography: island area. These findings underline the importance of paleogeography and relaxation dynamics for island communities, and of taking a geographically dynamic view to island biogeography.

The findings that overdispersion of insular Greek squamate communities increases with time‐since‐isolation and that landbridge islands have significantly higher PD NRI is consistent with the process of competitive exclusion driving the selective extinction of species in insular relaxation communities. Phylogenetic and functional community ecology offers two competing hypotheses for which process drives community structuring in relaxation communities, given extinction of species is non‐random and the functional traits considered are phylogenetically conserved (Haydon et al. 1993; Kraft et al. 2007; Webb 2000; Webb et al. 2002). Clustered assemblages of closely related, ecologically similar species (low PD and FD NRI) result from preferential extinction of species poorly suited to present conditions, while overdispersed communities (high PD/FD NRI) arise from competition limiting similarity (Figure 1A; Haydon et al. 1993; Webb 2000; Webb et al. 2002). Under the null hypothesis, extinctions are random with respect to phylogeny and functional traits, resulting from truly random extinction processes, or a confounding combination of environmental filtering and competitive exclusion. On the Greek islands considered here, significantly phylogenetically structured communities are mostly overdispersed (18.4% of all islands) with much lower clustering (2.5% of all islands) (Figure 2). Considering island type, communities on long‐time isolated islands and on unfragmented islands are most phylogenetically and functionally overdispersed (Figures 2 and 3), while the least overdispersion is found on comparatively young landbridge islands (Figures 2 and 3). These results suggest that the PD NRI of relaxing insular squamate communities becomes increasingly negative over time. FD NRI follows a similar though non‐significant trend. Phylogenetic clustering follows an inverse pattern to overdispersion, with no clustering on long‐time isolated islands and the highest degree of phylogenetic clustering on landbridge islands. Phylogenetic clustering on landbridge islands likely reflects both the non‐random composition of assemblages at the onset of isolation, where closely related, ecologically similar species persist, and the fact that these islands retain a relatively large and diverse subset of the regional species pool used in NRI randomizations, which increases the likelihood of observed clustering. Furthermore, young landbridge islands are typically large and are hence more likely to have elevated habitat diversity (e.g., along different altitudinal zones) thereby accommodating a diversity of closely related taxa specializing on similar, but distinct habitats. OLS models confirm that NRI decreases with increasing temporal isolation, supporting selective extinction on landbridge islands and a shift toward overdispersion over time. These combined observations possibly reflect the legacy of pre‐isolation and pre‐fragmentation communities, which initially experienced weaker competitive pressures under larger habitat areas with greater resource availability. As landbridges submerged, these communities became confined to smaller islands, leading to conditions of supersaturation (Diamond 1972; Hammoud et al. 2021; Wilcox 1978). Continued island contraction diminished resources (Foufopoulos et al. 2011), intensifying competition, especially between closely related and functionally similar species. This in turn resulted in a bias toward extinctions among closely related and presumably ecologically similar taxa, thus causing unstructured communities to become increasingly overdispersed as relaxation proceeded.

An example of a bias toward extinctions among phylogenetically related species is given by lacertid lizards. While on the Greek mainland up to six small‐bodied lacertid lizard species can occur in functional sympatry (e.g., *Algyroides moreoticus*, *Hellenolacerta graeca*, *Podarcis erhardii*, *P. ionicus*, *P. muralis* , *P. peloponnesiacus*, in the Basin of Feneos; Arnold 1987; Mayer et al. 1990), Greek landbridge islands rarely support more than two, despite these areas having been inhabited by similarly diverse mainland lizard communities pre‐fragmentation. Even more extreme, on long‐term isolated islands, none, with the sole exception of Rhodes (which only harbors *Ophisops elegans* and *Anatololacerta pelasgiana*), has more than a single small lacertid. For example, the giant island of Crete is inhabited only by *Podarcis cretensis*. As shown in earlier studies (Foufopoulos et al. 2011; Foufopoulos and Ives 1999), island extinctions in the region follow predictable patterns, with taxa going extinct on islands of decreasing size and age. Typically, the species that disappear first are those that have the smallest overall population size, that is, those that occur in naturally low population densities or are habitat specialists. Beyond this, taxa that have mainland ecological preferences for typical island habitats, which tend to be warmer and drier than nearby mainland regions, also have higher probabilities of surviving on islands post‐fragmentation. For example, among the aforementioned 6 species of lacertid lizards that occur syntopically on the Peloponnese, only one, *P. erhardii*, which on the mainland has a marginal existence on very dry, stony habitats, survives on the nearby Aegean islands. There however, it experiences ecological niche expansion as well as density inflation, and becomes ubiquitous colonizing even wet areas that it avoids on the mainland (Blondel et al. 1988). Whether ecological niche expansion and density inflation are primarily the result of reduced interspecific competition following extinction, or whether they in turn drive further competitive exclusion of surviving co‐occurring species, remains an open and compelling question.

Our findings are broadly consistent with patterns reported for Mediterranean‐wide squamate assemblages by Escoriza (2020), who similarly identified non‐random phylogenetic structure across islands and interpreted overdispersion as resulting from competitive processes. However, while Escoriza (2020) examined variation in assemblage structure in relation to island area, geographic isolation, and environmental gradients, our study explicitly incorporates temporal dynamics associated with sea‐level change. By linking community structure to paleo‐area, fragmentation history, and the temporal process of relaxation, we extend this understanding to show how community structure may predictably shift through time following isolation. These results highlight the importance of integrating temporal dynamics into island biogeography and call for further empirical testing.

Other work offers contrasting evidence of environmental filtering influencing squamate community assembly. Zhang et al. (2022) found that selective extinction and habitat nestedness influence squamate nestedness on landbridge islands in the Zhousan archipelago, suggesting environmental conditions shaped their community structure. Ramm et al. (2018) assessed the importance of different structuring mechanisms for squamate communities in environmentally benign and stressful habitats across continental Africa. Using the same functional traits as our study, body size and microhabitat preferences, they examined phylogenetic and functional structure across biomes. They found opposing patterns across a continent‐wide climatic gradient, concluding that environmental filtering dominates in stressful habitats, while competitive exclusion prevails in benign environments, corroborating the “stress dominance hypothesis” (Coyle et al. 2014; Swenson and Enquist 2007; Weiher and Keddy 1995). On Greek islands, squamate communities consistently exhibit higher functional than phylogenetic diversity on average (Figure 3), resulting in fewer functionally structured communities (Figure 2, Figure S1). Considering additional traits relevant to competition and persistence, like those related to territoriality, diet, and metabolic rate, could clarify whether the reduced functional structure reflects limitations in our study design or a genuine (absence of) ecological pattern. Finally, incorporating paleoclimate, land‐use and habitat diversity data, though currently not all available at suitable temporal resolution, could further explain functional clustering.

Studies of community ecology run the risk of overinterpreting detected patterns as proof of process (Colwell and Winkler 1984; Connolly et al. 2017), since multiple processes can produce similar patterns (Cardillo et al. 2008; Mayfield and Levine 2010; Vamosi et al. 2009). We thus prefer to take a cautious tone and encourage further investigation to more confidently infer the processes driving the nonrandom patterns presented here. Mechanistic modeling could explicitly test whether competitive exclusion drives extinctions in these relaxation communities (Connolly et al. 2017; Figueiredo et al. 2019). Such an approach could clarify whether local extinctions on Greek islands have structurally occurred within or between functional groups. If extinction removes lineages from islands while retaining the same number of functional groups, it would strongly support the competitive exclusion hypothesis proposed here. Including demographic information such as abundance estimates could also strengthen connections to formal coexistence theory (Kraft and Ackerly 2010). The presence/absence data used here omit ecological details linked to dominance and rarity, which are particularly important in species‐rich communities where fewer species are excluded and abundances hold most of the assembly information (Freilich and Connolly 2015; Münkemüller et al. 2012). Furthermore, while our analyses reveal patterns consistent with competition‐driven overdispersion, we note that we lack fine‐scale, within‐island distribution data. As a result, we cannot directly assess whether co‐occurring species are truly sympatric or spatially segregated, which would provide a more definitive test of ongoing competitive interactions. Despite these limitations, the patterns uncovered are compelling and merit further scrutiny, since they have limited ecologically consistent interpretations and exclude an important role of environmental filtering in relaxation on these islands. Finally, legacies of competitive exclusion could be underestimated due to the “Narcissus effect,” where the species pool is itself a reflection of competitive interactions shaping species coexistence, leading to systematic underestimation from biases inherent in the species pool (Colwell and Winkler 1984).

The results presented here highlight the formative role of past geographic change on contemporary island communities of squamates. The diversity of paleogeographic histories and responses to sea‐level rise of Greek islands, revealed by a detailed coastline model, provides an excellent system to study these effects. Patterns of phylogenetic community structure suggest that relaxation is phylogenetically selective, leaving communities more overdispersed than expected by chance—consistent with competitive exclusion, rather than environmental filtering, driving extinction. Time‐since‐isolation emerges as the strongest predictor of phylogenetic diversity, emphasizing the importance of legacy effects from glacial–interglacial geographic changes in shaping community composition. Understanding the mechanisms behind local extinction, coexistence, and equilibrium dynamics will aid conservation on islands, which are disproportionately affected by increasing global human impact (Fernández‐Palacios et al. 2021; Whittaker et al. 2017). These findings invite future research toward a mechanistic understanding of relaxation on Greek islands and beyond, integrating past environmental variability. By linking geographic history to contemporary biodiversity patterns, this study underscores the value of integrative approaches that combine community ecology, paleogeography, and conservation biology. Expanding this research to other archipelagos will help generalize principles of community relaxation and advance our understanding of how legacy effects shape ecosystems worldwide. A deeper understanding of extinction debt is critical, as actions taken today will determine the future genetic and ecological integrity of the countless species currently facing delayed extinction.

## Author Contributions

**Nathan M. Michielsen:** conceptualization (lead), data curation (lead), formal analysis (lead), investigation (lead), methodology (lead), visualization (lead), writing – original draft (lead), writing – review and editing (lead). **Cyril Hammoud:** investigation (supporting), writing – original draft (supporting), writing – review and editing (supporting). **Johannes Foufopoulos:** conceptualization (equal), data curation (equal), investigation (supporting), supervision (supporting), writing – original draft (supporting), writing – review and editing (supporting). **Kostas Kougioumoutzis:** writing – original draft (supporting), writing – review and editing (supporting). **Amandine Vidal‐Hosteng:** writing – original draft (supporting), writing – review and editing (supporting). **E. Emiel van Loon:** conceptualization (supporting), data curation (supporting), formal analysis (supporting), investigation (supporting), methodology (supporting), supervision (supporting), writing – original draft (supporting), writing – review and editing (supporting). **Johannes De Groeve:** data curation (supporting), formal analysis (supporting), investigation (supporting), methodology (supporting), software (supporting), writing – original draft (supporting), writing – review and editing (supporting). **Kenneth F. Rijsdijk:** conceptualization (equal), formal analysis (equal), investigation (equal), methodology (equal), supervision (equal), writing – original draft (equal), writing – review and editing (equal).

## Funding

This work was supported by Stichting Bevordering Herpetologie (SBH), Amsterdams Universiteitsfonds.

## Conflicts of Interest

The authors declare no conflicts of interest.

## Supporting information


**Figure S1:** Maps of study islands' island typology and multiple dimensions of squamate diversity. Subpanels show maps of the study region's current geography and reconstructed geography at the Last Glacial Maximum (LGM). Subpanels include information on (A) island typology in discrete colors (green = fragmented continental, purple = fragmented land bridge, pink = unfragmented continental, blue = unfragmented land bridge, orange = volcanic), (B) species richness in shades of blue, (C) phylogenetic diversity in shades of orange, and (D) functional diversity in shades of green. Outlines indicate significantly clustered communities; dotted outlines indicate significantly overdispersed communities.
**Figure S2:** Spatial distribution of functional traits across contemporary Aegean island reptile communities. (A) Community‐weighted mean (CWM) body size (log_10_ g) of squamate reptile assemblages across contemporary study islands, calculated as the mean log_10_‐transformed body mass of all species present on each island. (B) Percentage contribution of each microhabitat use category (saxicolous, terrestrial, arboreal, fossorial, aquatic, and generalist) to island squamate communities. For each island, the proportional representation of each microhabitat category was calculated as the fraction of species assigned to that category relative to total community size, with species assigned to compound microhabitat categories (e.g., terrestrial/saxicolous) contributing fractionally and equally to each constituent category. Gray polygons indicate islands present in the study region but lacking community data. Body size data from Feldman et al. (2016).
**Figure S3:** Correlation matrix of paleogeographic variables. Matrix shows pairwise correlation between paleogeographic predictor variables. The more positive and darker blue, the more strongly positively correlated respective variables are; the more negative and darker red, the more strongly negatively correlated respective variables are.
**Figure S4:** Effect of paleogeographic variables on diversity metrics. Biplots show univariate relationships between significant continuous variables per diversity metric. Regression lines indicate predictions from linear models, shaded areas indicate standard errors. Shading of points indicate community structure in respective island communities for PD and FD, with light gray indicating significant overdispersion, white indicating no significant structure and dark gray indicating significant clustering.
**Figure S5:** Coefficients of categorical variables in preferred models. Horizontal bars indicate the directions and scaled effect sizes of significant categorical parameters of preferred OLS models (Table 2) per diversity metric. Arrow indicates if direction of effect increases phylogenetic and functional overdispersion or clustering.
**Table S1:** Summary statistics. Table contains summary statistics of all response variables and continuous predictor variables, per island type (groups based on combination of geologic origin and geographic response to sea level rise and solely based on geographic response to sea level rise) and for all islands combined. Note that although for island typology fragmentation was not taken into account for volcanic islands, it was taken into account when solely designating the fragmentation history of islands. Per variable, the minimum and maximum observed values are given. Mean values and standard deviations are given between parentheses. SR = species richness, PD NRI = phylogenetic NRI, FD NRI = functional NRI. For diversity metrics, significant difference in means was assessed between groups within rows with similar shading (Tables S3 and S4). Significant differences in means between groups are indicated by mutually exclusive letters in superscript (*p* < 0.05, one‐way ANOVA with Tukey HSD post hoc test if number of groups was greater than two, otherwise two‐tailed *t*‐test, Tables S3 and S4).
**Table S2:** Island Species Area Relationships (ISAR), Island Phylogenetic Diversity Area Relationships (IPDAR) and Island Functional Diversity Area Relationships (IFDAR). Table contains model summaries of ISARs, IPDARs and IFDARs of squamates on study islands. Preferred functions are indicated by asterisks and significant *p*‐values are in bold.
**Table S3:** Two tailed *t*‐test results. Table contains *p* values of two tailed *t*‐tests of differences in means of diversity metrics between island types based solely on geographic response to sea level rise. Significantly different groups in bold.
**Table S4:** Tukey HSD post hoc test of diversity metrics per island type. Table contains adjusted *p* values of pairwise comparisons of island types' based on the combination geologic origin and geographic response to sea level rise mean species richness (SR), phylogenetic NRI (PD NRI), and functional NRI (FD NRI) based on Tukey HSD post hoc tests of one‐way ANOVA models. Significantly different pairs (*p* < 0.05) are in bold.
**Table S5:** Phylogenetic signal of squamate body size. Table contains *K*‐statistic of phylogenetic signal test of squamate body size and mean observed variance, *z* score, and *p* value based on observed variance of phylogenetically independent contrasts relative to 999 tip shuffling randomizations.

**Data S1:** ece373894‐sup‐0002‐DataS1.csv.

**Data S2:** ece373894‐sup‐0003‐DataS2.csv.

## Data Availability

All raw data and code to reproduce the analyses and figures in this paper are publicly available in online data repositories. Paleogeography data set: https://doi.org/10.21942/uva.c.6678104.v1. Per island species list data set: https://doi.org/10.21942/uva.23284868.v2. Data and code to reproduce analyses and figures: https://doi.org/10.6084/m9.figshare.29009042.
