# Supplementary material for: Who Is Paying the Extinction Debt? Phylogenetic and Functional Structuring on Greek Islands Is Shaped by Sea‐Level Rise Since the Last Glacial Maximum
Source: Ecol Evol. 2026 Jun 29;16(7):e73894. doi: 10.1002/ece3.73894 (PMC13314317; doi:10.1002/ece3.73894)
Supplement: Supplementary file 1 — Figure S1: Maps of study islands' island typology and multiple dimensions of squamate diversity. Subpanels show maps of the study region's current geography and reconstructed geography at the Last Glacial Maximum (LGM). Subpanels include information on (A) island typology in discrete colors (green = fragmented continental, purple = fragmented land bridge, pink = unfragmented continental, blue = unfragmented land bridge, orange = volcanic), (B) species richness in shades of blue, (C) phylogenetic diversity in shades of orange, and (D) functional diversity in shades of green. Outlines indicate significantly clustered communities; dotted outlines indicate significantly overdispersed communities. Figure S2: Spatial distribution of functional traits across contemporary Aegean island reptile communities. (A) Community‐weighted mean (CWM) body size (log10 g) of squamate reptile assemblages across contemporary study islands, calculated as the mean log10‐transformed body mass of all species present on each island. (B) Percentage contribution of each microhabitat use category (saxicolous, terrestrial, arboreal, fossorial, aquatic, and generalist) to island squamate communities. For each island, the proportional representation of each microhabitat category was calculated as the fraction of species assigned to that category relative to total community size, with species assigned to compound microhabitat categories (e.g., terrestrial/saxicolous) contributing fractionally and equally to each constituent category. Gray polygons indicate islands present in the study region but lacking community data. Body size data from Feldman et al. (2016). Figure S3: Correlation matrix of paleogeographic variables. Matrix shows pairwise correlation between paleogeographic predictor variables. The more positive and darker blue, the more strongly positively correlated respective variables are; the more negative and darker red, the more strongly negatively correlated respective variables are. Figur [file ECE3-16-e73894-s001.docx]

**Supplementary Information for:**

**Who is paying the extinction debt? Phylogenetic and functional structuring on Greek islands shaped by post-LGM sea level rise**

Nathan M. Michielsen^1,2,3^, Cyril Hammoud^4^, Johannes Foufopoulos^5^, Kostas Kougioumoutzis^6^, Amandine Vidal-Hosteng^7,8^, E. Emiel van Loon^1^, Johannes De Groeve^1^, Kenneth F. Rijsdijk^1^

^1^ Institute for Biodiversity and Ecosystem Dynamics, University of Amsterdam, Amsterdam, the Netherlands

^2^The Environment Institute, School of Biological Sciences, Adelaide University, Adelaide SA, 5005, Australia

^3^Center for Macroecology, Evolution, and Climate, Globe Institute, University of Copenhagen, Copenhagen Ø 2100, Denmark

^4^Department of Coastal Systems, Royal Netherlands Institute for Sea Research, Den Burg, The Netherlands.

^5^School for Environment and Sustainability, University of Michigan, Ann Arbor, MI 48109, USA

^6^Laboratory of Botany, Division of Plant Biology, Department of Biology, University of Patras, GR 26504 Patras, Greece

^7^Centre de Recherche sur la Biodiversité et l’Environnement, Université de Toulouse, Toulouse, France

^8^Groningen Institute For Evolutionary Life Sciences, University of Groningen, Groningen, The Netherlands

*Ecology and Evolution*

**Includes:**

Supplementary note on the natural history of Aegean squamate communities

Supplementary Figures S1 - S4

Supplementary Tables S1 - S5

Supplementary References

**Supplementary note on the natural history of Aegean squamate communities**

The squamate fauna of the Aegean islands is composed of lizards and snakes. Lizards, represented primarily by species in the genera *Podarcis*, *Lacerta*, and *Mediodactylus*, are predominantly invertebrate predators and occur in numerically dense populations. Snakes, including species of *Dolichophis*, *Elaphe*, *Natrix*, and *Vipera*, are predominantly vertebrate predators and occur at considerably lower densities. Although tortoises (*Testudo*) and terrapins (*Mauremys*) form part of the broader reptile fauna of the Aegean islands, they are not considered in the present study.

Situated at the nexus of three continents, the squamate fauna of the Aegean reflects multiple biogeographic source areas, and species composition differs markedly between island clusters according to the nearest mainland. For example, the islands of the Dodecanese are inhabited predominantly by species of Asian and African origin, while on the Ionian and Sporades islands, taxa are of predominantly European origin. The region harbours a pronounced endemic element, including *Podarcis milensis*, *P. cretensis*, *P. gaigeae*, *Mediodactylus oertzeni*, *Macrovipera schweizeri*, and *Dolichophis jugularis zinneri*, which, as expected given the dispersal constraints outlined in the main text, are largely restricted to pre-Pleistocene deep-water islands. Species communities across the archipelago are highly nested, with smaller and more isolated islands harbouring only subsets of the species found on larger islands. Squamate extinction in the region follows a predictable pattern, with species disappearing in a sequence based on relative population size: habitat specialists, low-density taxa, and heat/aridity-intolerant species go extinct first.

**Supplementary Figures**


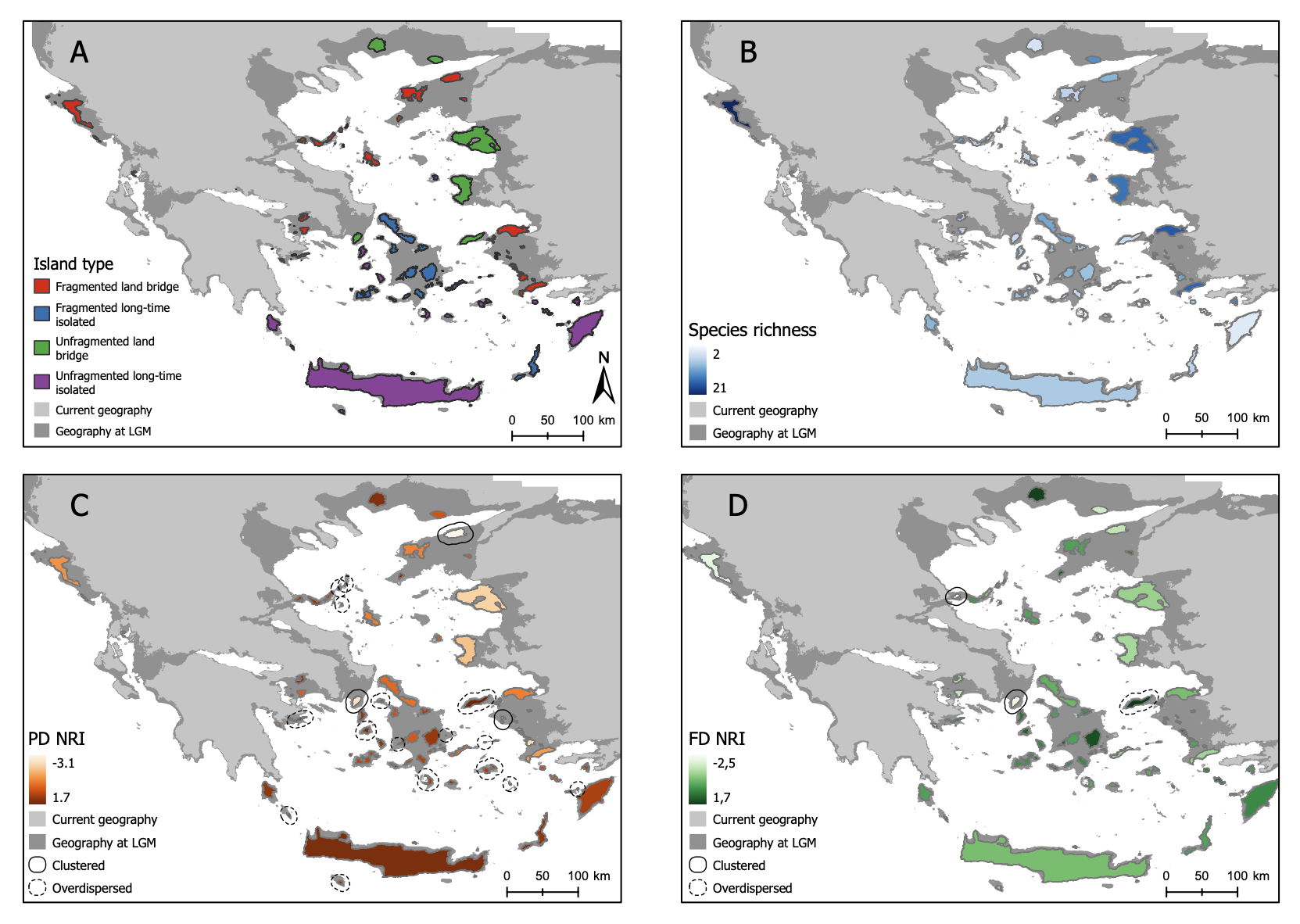


**Figure S1 – Maps of study islands’ island typology and multiple dimensions of squamate diversity.** Subpanels show maps of the study region’s current geography and reconstructed geography at the Last Glacial Maximum (LGM). Subpanels include information on (A) island typology in discrete colors (green = fragmented continental, purple = fragmented land bridge, pink = unfragmented continental, blue = unfragmented land bridge, orange = volcanic), (B) species richness in shades of blue, (C) phylogenetic diversity in shades of orange, and (D) functional diversity in shades of green. Outlines indicate significantly clustered communities; dotted outlines indicate significantly overdispersed communities.

**
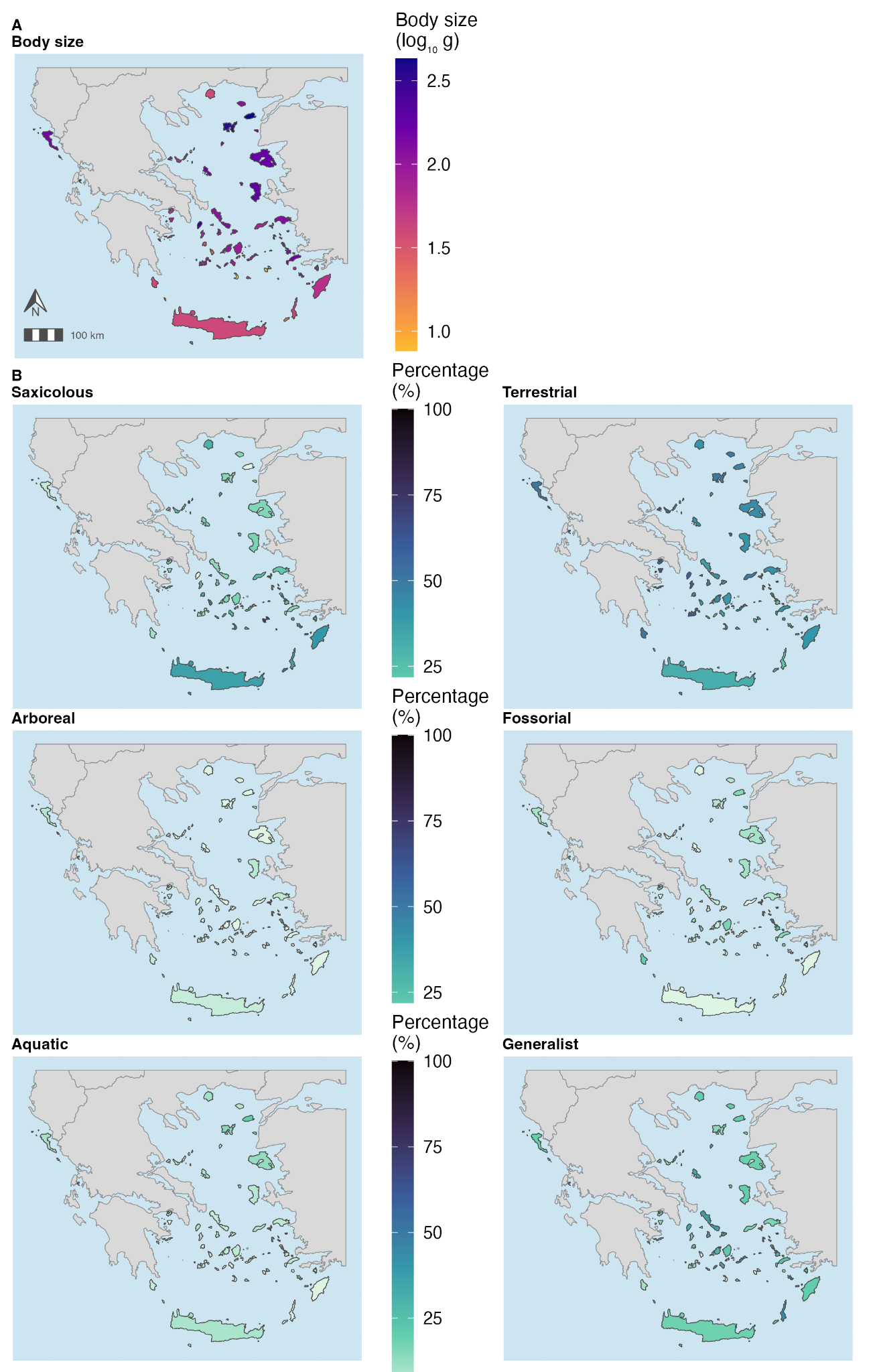
**

**Figure S2 – Spatial distribution of functional traits across contemporary Aegean island reptile communities**. (A) Community-weighted mean (CWM) body size (log_10_ g) of squamate reptile assemblages across contemporary study islands, calculated as the mean log_10_-transformed body mass of all species present on each island. (B) Percentage contribution of each microhabitat use category (saxicolous, terrestrial, arboreal, fossorial, aquatic, and generalist) to island squamate communities. For each island, the proportional representation of each microhabitat category was calculated as the fraction of species assigned to that category relative to total community size, with species assigned to compound microhabitat categories (e.g. terrestrial/saxicolous) contributing fractionally and equally to each constituent category. Grey polygons indicate islands present in the study region but lacking community data. Body size data from Feldman et al. [(2016)](https://www.zotero.org/google-docs/?QhkA0J).

**
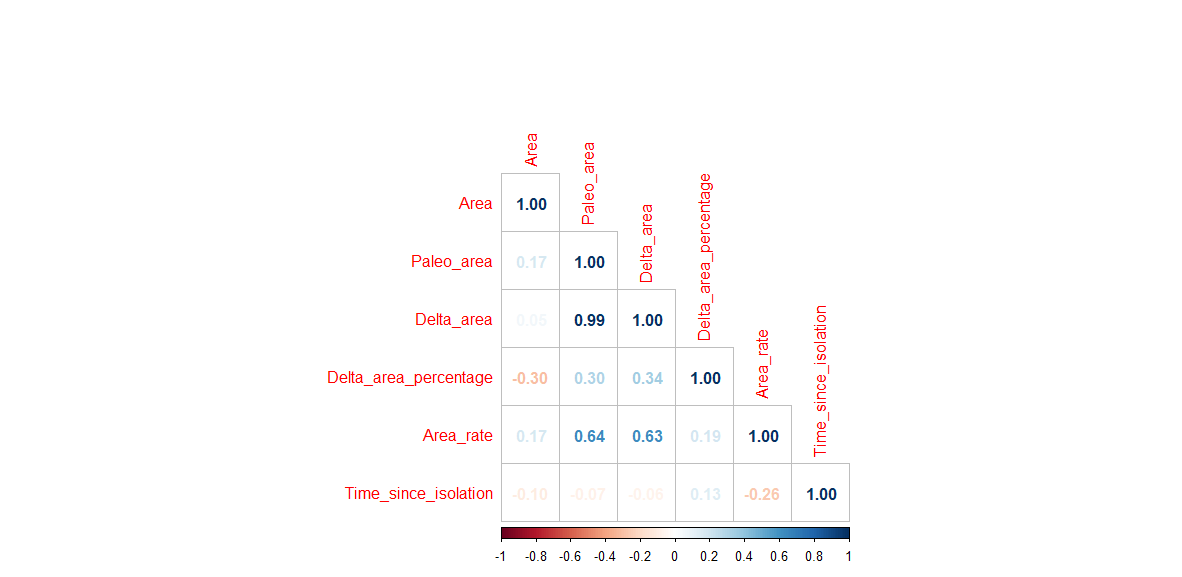
**

**Figure S3 – Correlation matrix of paleogeographic variables.** Matrix shows pairwise correlation between paleogeographic predictor variables. The more positive and darker blue, the more strongly positively correlated respective variables are; the more negative and darker red, the more strongly negatively correlated respective variables are.


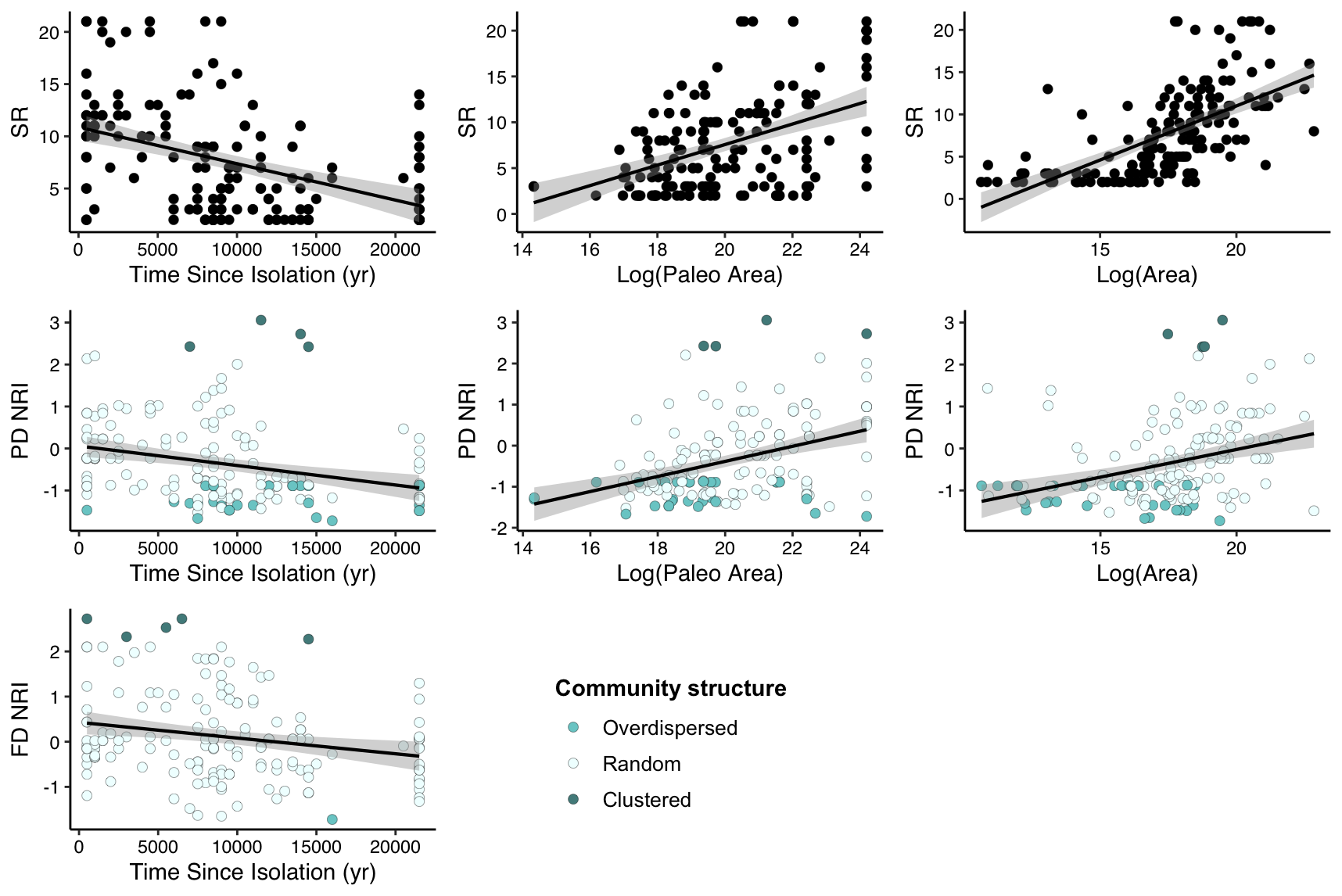


**Figure S4 – Effect of paleogeographic variables on diversity metrics.** Biplots show univariate relationships between significant continuous variables per diversity metric. Regression lines indicate predictions from linear models, shaded areas indicate standard errors. Shading of points indicate community structure in respective island communities for PD and FD, with light gray indicating significant overdispersion, white indicating no significant structure and dark gray indicating significant clustering.


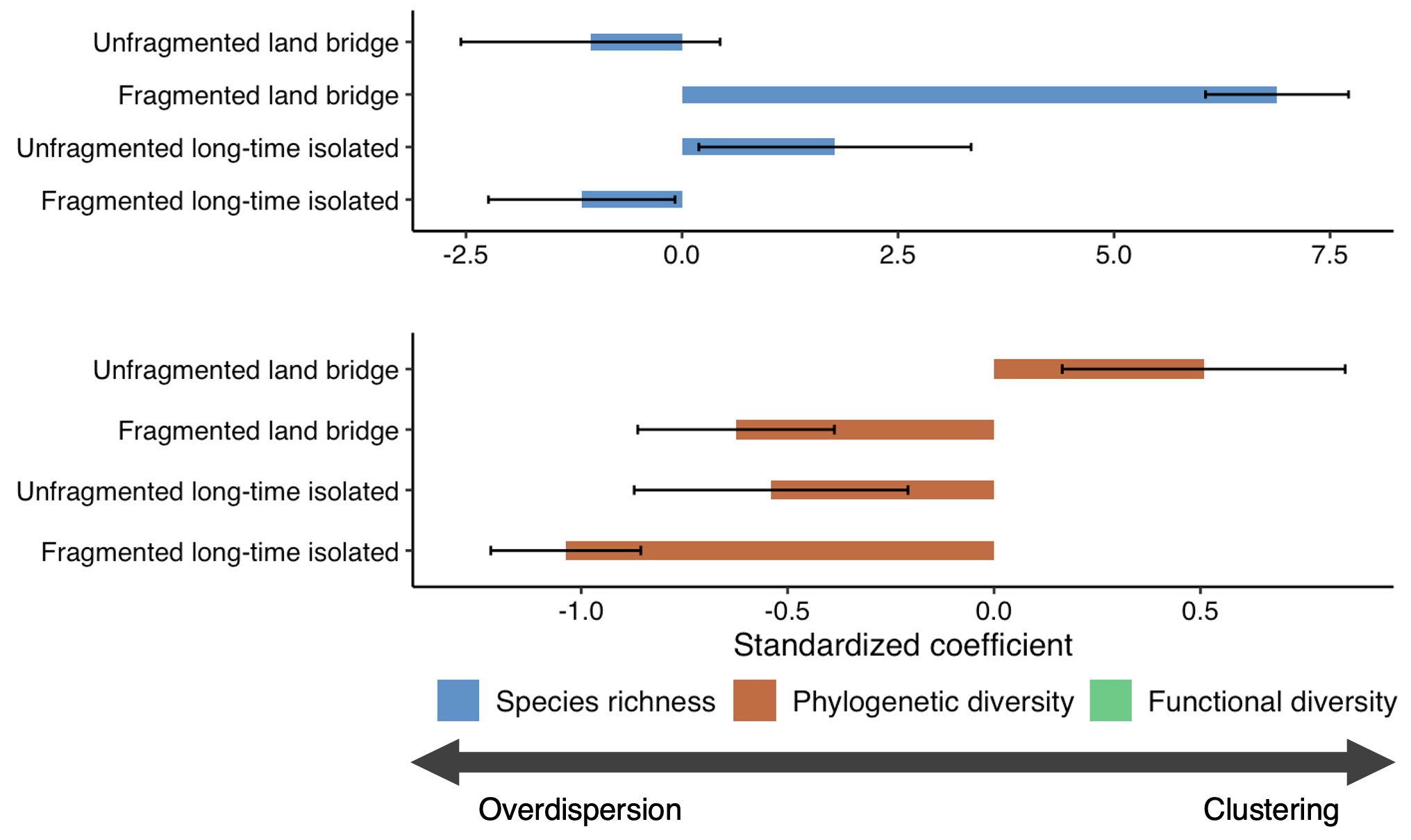


**Figure S5 – Coefficients of categorical variables in preferred models.** Horizontal bars indicate the directions and scaled effect sizes of significant categorical parameters of preferred OLS models (Table 2) per diversity metric. Arrow indicates if direction of effect increases phylogenetic and functional overdispersion or clustering.

# **Supplementary Tables**

**Table S1 – Summary statistics.** Table contains summary statistics of all response variables and continuous predictor variables, per island type (groups based on combination of geologic origin and geographic response to sea level rise and solely based on geographic response to sea level rise ) and for all islands combined. Note that although for island typology fragmentation was not taken into account for volcanic islands, it was taken into account when solely designating the fragmentation history of islands. Per variable, the minimum and maximum observed values are given. Mean values and standard deviations are given between parentheses. SR = species richness, PD NRI = phylogenetic NRI, FD NRI = functional NRI. For diversity metrics, significant difference in means was assessed between groups within rows with similar shading (Table S3 and S4). Significant differences in means between groups are indicated by mutually exclusive letters in superscript (p < 0.05, one-way ANOVA with Tukey HSD post-hoc test if number of groups was greater than two, otherwise two-tailed t-test, Table S3 and S4).

| **Island type** | **n** | **SR** | **PD NRI** | **FD NRI** |
| --- | --- | --- | --- | --- |
| Fragmented long-time isolated | 69 | 6.8 ± 3.6 (2 – 13) ^a^ | -0.7 ± 0.6 (-1.7 – 0.8) ^a^ | -0.2 ± 0.6 (-1.6 – 1.9) ^a^ |
| Fragmented land bridge | 63 | 9.0 ± 6.6 (2 – 21) ^a^ | 0 ± 1.0 (-1.5 – 3.1) ^bc^ | 0.6 ± 1.1 (-1.3 – 2.7) ^b^ |
| Unfragmented long-time isolated | 22 | 6.2 ± 4.3 (2 – 16) ^a^ | -0.7 ± 0.8 (-1.5 – 2.1) ^a^ | -0.3 ± 0.7 (-1.3 – 1.3) ^a^ |
| Unfragmented land bridge | 9 | 8.7 ± 4.6 (5 – 16) ^a^ | 0.3 ± 1.9 (-1.7 – 2.7) ^bc^ | 0.4 ± 1.4 (-1.7 – 2.3) ^ab^ |
| All land bridge | 72 | 8.9 ± 6.3 (2 – 21) ^a^ | 0.1 ± 1.2 (-1.7 – 3.1) ^a^ | 0.6 ± 1.1 (-1.6 – 2.7) ^a^ |
| All long-time isolated | 91 | 6.6 ± 3.8 (2 – 16) ^a^ | -0.7 ± 0.6 (-1.7 – 2.1) ^b^ | -0.3 ± 0.6 (-1.7 – 1.9) ^b^ |
| All fragmented | 132 | 7.8 ± 5.3 (2 – 21) ^a^ | -0.4 ± 0.9 (-1.7 – 3.1) ^a^ | 0.2 ± 1.0 (-1.6 – 2.7) ^a^ |
| All unfragmented | 31 | 6.9 ± 4.5 (2 – 16) ^a^ | -0.4 ± 1.3 (-1.7 – 2.7) ^a^ | -0.1 ± 1.0 (-1.7 – 2.3) ^a^ |
| All combined | 163 | 7.7 ± 5.2 (2 – 21) | -0.4 ± 1.0 (-1.7 – 3.1) | 0.1 ± 1.0 (-1.7 – 2.7) |

| **Island type** | **Area (m^2^)** | **Paleo area (m^2^)** | **Time since isolation (yr)** |
| --- | --- | --- | --- |
|  |  |  |  |
| Fragmented long-time isolated | 3.1*10^8^ ± 8.0*10^8^ (5.4*10^4^ – 5.9*10^9^) | 1.1*10^9^ ± 1.8*10^9^ (1.7*10^6^ – 7.0*10^9^) | 7529 ± 5001 (500 – 20500) |
| Fragmented land bridge | 1.6*10^8^ ± 2.9*10^8^ (4.2*10^4^ – 1.7*10^9^) | 5.6*10^9^ ± 1.0*10^10^ (3.5*10^7^ - 3.2*10^10^) | 6867 ± 4161 (500 – 14500) |
| Unfragmented long-time isolated | 8.2*10^8^ ± 2.3*10^9^ (3.5*10^6^ – 8.3*10^9^) | 1.1*10^9^ ± 2.7*10^9^ (2.4*10^7^ – 1.1*10^10^) | 19909 ± 5261 (500 – 21500) |
| Unfragmented land bridge | 3.9*10^8^ ± 5.4*10^8^ (2.5*10^6^ – 1.7*10^9^) | 1.6*10^10^ ± 1.6*10^10^ (2.7*10^8^ – 3.2*10^10^) | 12056 ± 3046 (9000 – 16000) |
| All land bridge | 1.9*10^8^ ± 3.4*10^8^ (4.2*10^4^ – 1.7*10^9^) | 6.8*10^9^ ± 1.2*10^10^ (3.5*10^6^ -3.2*10^10^) | 7507 ± 4378 (500 – 16000) |
| All long-time isolated | 4.3*10^8^ ± 1.3*10^9^ (5.4*10^4^ -8.3*10^9^) | 1.1*10^9^ ± 2.0*10^9^ (1.7*10^7^ -1.1*10^10^) | 10522 ± 7332 (500 – 21500) |
| All fragmented | 2.4*10^8^ ± 6.2*10^8^ (4.2*10^4^ -5.9*10^9^) | 3.2*10^9^ ± 7.6*10^9^ (1.7*10^6^ -3.2*10^10^) | 7208 ± 4614 (500 – 20500) |
| All unfragmented | 6.9*10^8^ ± 1.9*10^9^ (2.5*10^6^ -8.3*10^9^) | 5.3*10^9^ ± 1.1*10^10^ (2.4*10^7^ -3.2*10^10^) | 17629 ± 5915 (500 – 21500) |
| All combined | 3.2*10^8^ ± 1.0*10^9^ (4.2*10^6^ – 8.8*10^9^) | 3.6*10^9^ ± 8.3*10^9^ (1.7*10^6^ – 3.2*10^10^) | 9190 ± 6366 (500 – 21500) |

**Table S2 – Island Species Area Relationships (ISAR), Island Phylogenetic Diversity Area Relationships (IPDAR) and Island Functional Diversity Area Relationships (IFDAR) .** Table contains model summaries of ISARs, IPDARs and IFDARs of squamates on study islands. Preferred functions are indicated by asterisks and significant p-values are in bold.

| Form | Function | p-value | Adjusted R^2^ | |
| --- | --- | --- | --- | --- |
| Linear | SR = c + z * A | **< 0.001** | 0.074 |  |
| Power* | SR = c + A ^ z | **< 0.001** | 0.41 | |
| Exponential | SR = c + z * log(A) | **< 0.001** | 0.40 | |
| Linear | PD = c + z * A | **0.009** | 0.036 | |
| Power | PD = c + A ^ z | **< 0.001** | 0.072 | |
| Exponential* | PD = c + z * log(A) | **< 0.001** | 0.12 | |
| Linear | FD = c + z * A | 0.6 | -0.0046 | |
| Power | FD = c + A ^ z | **0.04** | 0.019 | |
| Exponential* | FD = c + z * log(A) | **0.02** | 0.030 | |

**Table S3 – Two tailed t-test results.** Table contains p values of two tailed t-tests of differences in means of diversity metrics between island types based solely on geographic response to sea level rise. Significantly different groups in bold.

| Diversity metric | Land bridge vs. long-time isolated islands  p value | Fragmented vs. unfragmented islands  p value |
| --- | --- | --- |
| SR | **0.006** | 0.319 |
| PD | **< 0.001** | 0.694 |
| FD | **< 0.001** | 0.295 |

**Table S4 – Tukey HSD post-hoc test of diversity metrics per island type.** Table contains adjusted p values of pairwise comparisons of island types’ based on the combination geologic origin and geographic response to sea level rise mean species richness (SR), phylogenetic NRI (PD NRI), and functional NRI (FD NRI) based on Tukey HSD post-hoc tests of one-way ANOVA models. Significantly different pairs (p < 0.05) are in bold.

| SR |  |  |  |  |
| --- | --- | --- | --- | --- |
|  | Fragmented long-time isolated | Fragmented land bridge | Unfragmented long-time isolated | Unfragmented land bridge |
| Fragmented long-time isolated | - |  |  |  |
| Fragmented land bridge | 0.056 | - |  |  |
| Unfragmented long-time isolated | 0.968 | 0.115 | - |  |
| Unfragmented land bridge | 0.714 | 0.997 | 0.606 | - |
| PD NRI |  |  |  |  |
|  | Fragmented long-time isolated | Fragmented land bridge | Unfragmented long-time isolated | Unfragmented land bridge |
| Fragmented long-time isolated | - |  |  |  |
| Fragmented land bridge | **< 0.001** | - |  |  |
| Unfragmented long-time isolated | 0.992 | **0.003** | - |  |
| Unfragmented land bridge | **0.015** | 0.794 | **0.013** | - |
| FD NRI |  |  |  |  |
|  | Fragmented long-time isolated | Fragmented land bridge | Unfragmented long-time isolated | Unfragmented land bridge |
| Fragmented long-time isolated | - |  |  |  |
| Fragmented land bridge | **< 0.001** | - |  |  |
| Unfragmented long-time isolated | 1.0 | **0.001** | - |  |
| Unfragmented land bridge | 0.142 | 0.977 | 0.196 | - |

**Table S5 – Phylogenetic signal of squamate body size.** Table contains K-statistic of phylogenetic signal test of squamate body size and mean observed variance, z score, and p value based on observed variance of phylogenetically independent contrasts relative to 999 tip shuffling randomizations.

| K-statistic | Mean observed variance | z score | p value |
| --- | --- | --- | --- |
| 0.80 | 0.009 | 0.042 | **0.001** |
